# Risk factors and effectiveness of vaccination for nosocomial SARS-CoV-2 acquisition throughout the SARS-CoV-2 pandemic

**DOI:** 10.1186/s12879-025-11349-9

**Published:** 2025-08-19

**Authors:** Nishi Dave, Suzanne D. van der Werff, Daniel Sjöholm, Johan Zetterqvist, Pontus Nauclér

**Affiliations:** 1 Department of Medicine, Solna, Division of Infectious Diseases, Karolinska Institutet, Stockholm, Sweden; 2 Department of Infectious Diseases, Karolinska University Hospital, Stockholm, Sweden; 3 Department of Medicine, Solna, Clinical Epidemiology Division, Karolinska Institutet, Stockholm, Sweden

**Keywords:** Nosocomial infection, SARS-CoV-2, Risk factors, Vaccine effectiveness, Nested case-control

## Abstract

**Background:**

Studies elucidating the risk factors for nosocomial SARS-CoV-2 infections or assessing effectiveness of vaccination on acquisition prevention throughout the pandemic period are scarce. Here, we aimed to identify individual- and care-related risk factors and study the association between vaccination and risk of infection for nosocomial SARS-CoV-2 infections.

**Methods:**

We performed a nested case-control study of patients aged ≥ 18 years hospitalised in Region Stockholm, between 1 March 2020 -15 November 2023. Each case was matched to up to 10 controls, on admission date, time period, and length of stay. Individual factors of sex, age, region of birth, education level, comorbidities and care-related factors such as number of transfers during care episode, surgery status, type of hospital unit and hospitals in the region were assessed. Vaccine effectiveness was assessed for number of doses and time since last vaccination. Conditional logistic regression was used to calculate odds ratio for risk factors and vaccine effectiveness.

**Results:**

Among 2711 cases and 27,065 matched controls, older age (90 + years: 4.11 [2.71–6.23]), male sex (1.11 [1.02–1.21]) and chronic lung disease (1.25 [1.12 to 1.40]) were associated with increased odds of nosocomial infection. Among care-related factors, admission to geriatric hospital units (1.54 [1.26–1.89]) and increased number of transfers (2 + transfers: 2.48 [1.89–2.34]) were associated with higher odds. Overall, while vaccination with 2 or more doses and any time since last dose compared to being unvaccinated was associated with lower odds of infections, we observed that vaccination with 3 doses (aOR: 0.55, 95% CI: 0.46–0.67), and those with their latest dose administered within the last 3 months had the lowest odds of infection (aOR: 0.48, 95% CI: 0.39 to 0.59).

**Conclusions:**

We demonstrated that vaccination is effective in reducing the risk of nosocomial SARS-CoV-2 infection, and preventive measures during early phases of pandemics should focus on high-risk patient groups. Risk of infection can be further reduced by focusing on high-risk areas within hospital settings and by reducing patient transfers during their care episode.

**Supplementary Information:**

The online version contains supplementary material available at 10.1186/s12879-025-11349-9.

## Introduction

Transmission of severe acute respiratory syndrome coronavirus 2 (SARS-CoV-2) in healthcare settings leads to increased morbidity and mortality [1, 2]. Nosocomial SARS-CoV-2 infections are associated with an increased risk of mortality when compared to patients with community-acquired SARS-CoV-2 infections, and both an increased mortality and prolonged length of stay (LOS) when compared to patients without nosocomial SARS-CoV-2 infections [1, 3, 4]. Identification of risk factors for nosocomial infections can help design strategic and effective measures to reduce transmission and risk of infection.

Studies on risk factors for nosocomial SARS-CoV-2 infections have primarily been conducted during the early phases of the pandemic, not accounting for the effect of the different types of variants or vaccination, have focused on single outbreaks, have been restricted to small sample size of cases or focused on single hospitals or healthcare centres [5–8]. Studies have identified that severity of SARS-CoV-2 infections was lower in the vaccination period [3, 9], however, these studies do not directly assess the effectiveness of vaccination to prevent nosocomial SARS-CoV-2 infections among patients.

The aim of this study was to identify individual and care-related risk factors associated with nosocomial SARS-CoV-2 infections among patients admitted to hospitals in Region Stockholm and specifically assess the association between vaccination status and risk of nosocomial infection.

## Methods

### Study design and population

We identified all individuals aged 18 years or older, residing in Region Stockholm, Sweden, for at least 3 years prior to the study start on March 1, 2020, to ensure reliable data on underlying comorbidities (*N* = 2094804). In this population we identified a cohort of 134,545 healthcare admissions among 87,041 patients between March 1, 2020, to November 15, 2023, with a LOS > 7 days, in which we conducted a nested matched case-control study.

Patients testing polymerase chain reaction (PCR) positive for SARS-CoV-2 were classified as having a nosocomial infection if the test was performed on day 8 or later after admission or up to 2 days after discharge, given a LOS of at least 8 days [3, 10]. Each patient with a nosocomial SARS-CoV-2 infection, hereafter defined as case, was matched to up to ten patients without a confirmed infection, hereafter referred to as controls, using sampling with replacement. Matching criteria were time period (pre-vaccination, period 1 or period 2), calendar time of admission (controls to be admitted to a hospital ± 10 days within admission date of case) and time spent in hospital until the index date, to ensure that cases and controls had the same LOS (risk period) up until that date. Index date for the cases was the date of the PCR positive SARS-CoV-2 test and for the controls, the date of the admission for the control plus the days until positive PCR test result for the matched case. All analyses were restricted to the first PCR positive SARS-CoV-2 test for every case.

Patients were excluded if they were admitted with a main diagnosis of COVID-19 or had admissions with LOS > 90 days corresponding to atypical clinical profiles that might introduce bias.

Three time periods accounting for epidemic and vaccination variation across the study period were defined. The pre-vaccination period was from March 1, 2020, with the end date defined as 4 weeks after the first public campaign of SARS-CoV-2 vaccination, i.e., until January 24, 2021. Based on information on SARS-CoV-2 variant distribution, two post-vaccination periods were defined: period 1 from January 25, 2021, until January 6, 2022; and period 2 (Omicron period) from January 7, 2022, until November 15, 2023, where the Omicron variant accounted for at least 90% of all sampled sequences in Stockholm County [3].

The study was approved by the Swedish Ethical Review Board.

### Data sources

Data from various registers were used in this study, with information being linked using personal identification numbers unique to all residents in Sweden. Information on positive PCR test results for SARS-CoV-2 were obtained from the register of notifiable diseases in Sweden, called SmiNet [11]. Data on cycle threshold (ct) values for PCR tests was obtained from the Quality Register for SARS-CoV-2, containing information from laboratory systems [12]. Vaccination information for all participants was obtained from the national vaccination register of the Public Health Agency of Sweden [13]. Information on patient admissions, patient demographic characteristics and comorbidities were obtained from the Region Stockholm Healthcare Data Warehouse (VAL) database [14]. Data on educational level and region of birth were obtained from the Longitudinal Integrated Database for Health Insurance and Labour Market Studies database, Statistics Sweden [15].

### Exposure variables

The following individual-related exposures were analysed: sex (men or women), age groups (18–30, 31–40, 41–50, 51–60, 61–70, 71–80, 81–90, 90 + years), region of birth (Sweden, Africa, Europe, The Americas, Asia and Oceania), education level (Primary, Secondary, Tertiary), comorbidities based on the ICD-10 codes grouped as cancer, cardiovascular diseases, chronic kidney diseases, chronic lung diseases, diabetes, hypertension, immunosuppression (eTable1), with the model adjusted for number of vaccine doses at index date (0, 1, 2, 3, 4, 5 + vaccine doses). Number of vaccine doses was modelled such that the last vaccine dose was administered 14 days prior to index date to account for the development of the protective effect and complete immune response [16].

Care-related factors were defined as: number of transfers during the care episode (0, 1, 2 + transfers), surgery status before index date, type of hospital unit (Geriatric, Haematology/Oncology/Transplant, Infection, Medicine, Psychiatric, Surgery, and Other), and hospitals in the region (Danderyd, Karolinska Huddinge, Karolinska Solna, South General, St Göran, Södertälje, Norrtälje, and Other). We included information on hospital to adjust for potential confounding due to differences in structural changes, infection prevention practices and patient populations. Hospital units focus on care areas where patients are admitted for acute or subacute care. For example, geriatric hospital units focus on management of care for older patients with complex health needs and are distinct to long-term care or nursing home settings. All care-related variables were assessed in the last 7 days up until index date to capture the timeframe for when patients would be exposed to SARS-CoV-2.

### Statistical analysis

Continuous data is presented using median and interquartile range (IQR); categorical data is presented as frequencies and percentages. For patients with complete data, risk factors for nosocomial SARS-CoV-2 infection were analysed using univariate and multivariate conditional logistic regression models. The multivariate models included age groups, sex, comorbidities, education level, region of birth, number of vaccine doses, surgery status before index date, number of transfers, hospital, and hospital units. Age groups, education level, region of birth, number of vaccine doses and number of transfers were modelled as categorical variables; sex, comorbidities and surgery status were modelled as binary variables. To account for patients moving between different care facilities during their care episode, each hospital and hospital unit were coded as independent binary variables. Multicollinearity between the variables was assessed using the variance inflation factor [17].

Supplementary analyses were conducted for patients aged 65 years and older, stratified by time periods and to account for residual viral RNA from previous acute infections, we restricted the matched dataset to include cases with ct value ≤ 30 and their respective matched controls. Lastly, a sensitivity analysis was performed excluding controls that later became cases to account for potential misclassification due to incubation period and symptom onset.

Association between vaccination and nosocomial infection was analysed during periods 1 and 2 using univariate and multivariate conditional logistic regression models. The multivariate models were adjusted for age as a continuous variable using restricted cubic splines, to account for the non-linear effect of age, sex, comorbidities, education level, region of birth, hospitals, and hospital units to account for force of infection reflecting the variation in background risk of exposure. Patients with one vaccine dose were excluded due to incomplete immunological response [18].

First, vaccination status was classified as 2 + vaccine doses (vaccinated with 2 or more doses) compared to 0 doses of vaccine (unvaccinated). Second, to study the association between number of doses and infection acquisition, we compared patients with 0 vaccine doses to those with 2, 3, 4, 5 + vaccine doses. Third, comparing those with 0 vs. 2 + vaccine doses, the association between vaccination time and nosocomial infections was analysed as time since last administered vaccine dose: <3 months, 3–6, 6–12, and 12 + months. In a sensitivity analysis, to account for the effect of the hospital and hospital unit where patients were admitted, a separate case-control sampling was performed, where hospital and hospital unit were added as matching variables. Additionally, the vaccination analysis was re-run including patients with 1 dose to evaluate whether their inclusion affected the overall results.

Analyses were performed using R v4.1.0 and statistical significance was set at two-sided *P* <.05.

## Results

Of the 87,041 patients identified, we excluded 1901 patients who had a LOS > 90 days and 9789 patients who had a main diagnosis of COVID-19 at admission. From this cohort, we identified 2711 cases of nosocomial SARS-CoV-2 infections between March 1, 2020, until November 15, 2023. The highest number of cases was observed in period 2 (*n* = 1654), followed by pre-vaccination period (*n* = 724) and period 1 (*n* = 333).

### Individual factors and underlying diseases associated with nosocomial SARS-CoV-2 infections

The matching yielded a dataset comprised of 2711 cases and 27,065 controls. Among the cases, the overall median age was 81 years compared to 74 years for the controls, with highest proportion of cases observed in age group 81–90 years (36.7%). This was similar in pre-vaccination and period 2, however in period 1, cases were slightly younger (median age 77 years), and the highest proportion of cases (28.8%) was observed in age group 71–80 years (Table 1). Among the cases across all three time periods, women represented the highest proportion (52.6% among cases for the entire study). The highest proportion of cases were born in Sweden (82.2%) followed by Europe (13.5%) and most cases had secondary level of education (44.5%). Hypertension was the most common comorbidity (64.8%) followed by cardiovascular diseases (43.9%). The median time to positive SARS-CoV-2 test from admission among cases was 16 days in period 1 and 13 days in pre-vaccination and period 2 (Table 1). Overall, 48.0% and 89.1% of cases had 2 + vaccine doses in period 1 and 2, respectively.

**Table 1:** Demographic characteristics and individual-related factors among cases^a^ and controls

	**Overall**		**Pre-Vaccination**		**Period 1**		**Period 2**	
	**Controls (*N*=27065)**	**Cases (*N*=2711)**	**Controls (*N*=7223)**	**Cases (*N*=724)**	**Controls (*N*=3321)**	**Cases (*N*=333)**	**Controls (*N*=16521)**	**Cases (*N*=1654)**
Age (in years)								
Median [IQR]	74 [58, 82]	81 [74, 87]	72 [54, 82]	82 [74, 88]	73 [57, 82]	77 [67, 86]	74 [60, 83]	81 [74, 88]
Age groups (in years)								
18-30	1400 (5.2%)	30 (1.1%)	477 (6.6%)	6 (0.8%)	192 (5.8%)	8 (2.4%)	731 (4.4%)	16 (1.0%)
31-40	1597 (5.9%)	47 (1.7%)	480 (6.6%)	11 (1.5%)	194 (5.8%)	13 (3.9%)	923 (5.6%)	23 (1.4%)
41-50	1849 (6.8%)	65 (2.4%)	585 (8.1%)	12 (1.7%)	239 (7.2%)	18 (5.4%)	1025 (6.2%)	35 (2.1%)
51-60	2774 (10.2%)	117 (4.3%)	794 (11.0%)	26 (3.6%)	344 (10.4%)	22 (6.6%)	1636 (9.9%)	69 (4.2%)
61-70	4126 (15.2%)	269 (9.9%)	1089 (15.1%)	79 (10.9%)	521 (15.7%)	44 (13.2%)	2516 (15.2%)	146 (8.8%)
71-80	7327 (27.1%)	781 (28.8%)	1789 (24.8%)	195 (26.9%)	889 (26.8%)	96 (28.8%)	4649 (28.1%)	490 (29.6%)
81-90	6096 (22.5%)	996 (36.7%)	1502 (20.8%)	272 (37.6%)	705 (21.2%)	93 (27.9%)	3889 (23.5%)	631 (38.2%)
90+	1896 (7.0%)	406 (15.0%)	507 (7.0%)	123 (17.0%)	237 (7.1%)	39 (11.7%)	1152 (7.0%)	244 (14.8%)
Sex								
Women	14083 (52.0%)	1425 (52.6%)	3800 (52.6%)	375 (51.8%)	1687 (50.8%)	173 (52.0%)	8596 (52.0%)	877 (53.0%)
Men	12982 (48.0%)	1286 (47.4%)	3423 (47.4%)	349 (48.2%)	1634 (49.2%)	160 (48.0%)	7925 (48.0%)	777 (47.0%)
Region of birth								
Sweden	21131 (78.1%)	2229 (82.2%)	5663 (78.4%)	575 (79.4%)	2560 (77.1%)	271 (81.4%)	12908 (78.1%)	1383 (83.6%)
Africa	689 (2.5%)	28 (1.0%)	203 (2.8%)	10 (1.4%)	92 (2.8%)	7 (2.1%)	394 (2.4%)	11 (0.7%)
Europe	3399 (12.6%)	366 (13.5%)	882 (12.2%)	111 (15.3%)	461 (13.9%)	44 (13.2%)	2056 (12.4%)	211 (12.8%)
The Americas	432 (1.6%)	25 (0.9%)	117 (1.6%)	8 (1.1%)	42 (1.3%)	1 (0.3%)	273 (1.7%)	16 (1.0%)
Asia and Oceania	1414 (5.2%)	63 (2.3%)	358 (5.0%)	20 (2.8%)	166 (5.0%)	10 (3.0%)	890 (5.4%)	33 (2.0%)
Education level								
Primary	6871 (25.4%)	727 (26.8%)	1891 (26.2%)	219 (30.2%)	869 (26.2%)	85 (25.5%)	4111 (24.9%)	423 (25.6%)
Secondary	11614 (42.9%)	1207 (44.5%)	3075 (42.6%)	301 (41.6%)	1440 (43.4%)	152 (45.6%)	7099 (43.0%)	754 (45.6%)
Tertiary	8580 (31.7%)	777 (28.7%)	2257 (31.2%)	204 (28.2%)	1012 (30.5%)	96 (28.8%)	5311 (32.1%)	477 (28.8%)
Time from admission to positive test								
Median [IQR]	0 [0, 0]	13 [10, 19]	0 [0, 0]	13 [10, 20.25]	0 [0, 0]	16 [11, 24]	0 [0, 0]	13 [10, 18]
Number of vaccine doses^b^								
0	10406 (38.4%)	1023 (37.7%)	N/A	N/A	1408 (42.4%)	146 (43.8%)	1776 (10.8%)	153 (9.3%)
1	653 (2.4%)	54 (2.0%)	N/A	N/A	260 (7.8%)	27 (8.1%)	392 (2.4%)	27 (1.6%)
2+	16006 (59.1%)	1634 (60.3%)	N/A	N/A	1653 (49.8%)	160 (48.0%)	14353 (86.9%)	1474 (89.1%)
Comorbidities^c^								
Cancer	4136 (15.3%)	503 (18.6%)	1318 (18.2%)	151 (20.9%)	522 (15.7%)	58 (17.4%)	2296 (13.9%)	294 (17.8%)
Cardiovascular diseases	8030 (29.7%)	1190 (43.9%)	2316 (32.1%)	381 (52.6%)	990 (29.8%)	145 (43.5%)	4724 (28.6%)	664 (40.1%)
Chronic kidney diseases	2590 (9.6%)	404 (14.9%)	811 (11.2%)	146 (20.2%)	350 (10.5%)	42 (12.6%)	1429 (8.6%)	216 (13.1%)
Chronic lung diseases	3190 (11.8%)	501 (18.5%)	900 (12.5%)	172 (23.8%)	428 (12.9%)	45 (13.5%)	1862 (11.3%)	284 (17.2%)
Diabetes	5474 (20.2%)	676 (24.9%)	1524 (21.1%)	212 (29.3%)	660 (19.9%)	73 (21.9%)	3290 (19.9%)	391 (23.6%)
Hypertension	13513 (49.9%)	1757 (64.8%)	3589 (49.7%)	475 (65.6%)	1653 (49.8%)	203 (61.0%)	8271 (50.1%)	1079 (65.2%)
Immunosuppression	1135 (4.2%)	91 (3.4%)	479 (6.6%)	37 (5.1%)	126 (3.8%)	12 (3.6%)	530 (3.2%)	42 (2.5%)

*Abbreviation*: *IQR* Interquartile range

^a^Includes patients with their first recorded SARS-CoV-2 infection

^b^Counted up until -14 days before index date

^c^Based on ICD-10 codes from -3 years to -30 days before start of study. See list of ICD-10 codes for each comorbidity category in eTable1

The adjusted conditional logistic regression analysis showed that those in age group 90 + years had the highest odds of acquiring an infection (aOR: 4.11, 95% CI: 2.71 to 6.23) (Table 2). Men (aOR: 1.11, 95% CI: 1.02 to 1.21) and patients with chronic lung diseases (aOR: 1.25, 95% CI: 1.12 to 1.40) had increased odds of nosocomial SARS-CoV-2 infections. Region of birth of Asia and Oceania were associated with decreased odds (aOR: 0.76, 95% CI: 0.58 to 0.99).

**Table 2:** Odds ratio for individual- and care-related risk factors of nosocomial SARS-CoV-2 infections comparing cases^a^ and controls

**Variable**	**Unadjusted**		**Adjusted**^**b**^	
	OR	95% CI	OR	95% CI
*Individual-related factors*				
Age groups (in years)				
18-30	Ref			
31-40	1.37	0.86 to 2.17	1.36	0.85 to 2.17
41-50	1.63	1.05 to 2.53	1.63	1.05 to 2.54
51-60	1.99	1.33 to 2.99	1.81	1.20 to 2.75
61-70	3.11	2.12 to 4.56	2.27	1.52 to 3.38
71-80	5.22	3.60 to 7.55	2.78	1.87 to 4.14
81-90	8.16	5.64 to 11.80	3.42	2.28 to 5.12
90+	10.91	7.47 to 15.94	4.11	2.71 to 6.23
Sex				
Women	Ref			
Men	0.98	0.90 to 1.06	1.11	1.02 to 1.21
Comorbidities				
Cancer	1.26	1.14 to 1.40	0.95	0.85 to 1.06
Cardiovascular diseases	1.87	1.72 to 2.03	1.07	0.98 to 1.18
Chronic kidney diseases	1.66	1.48 to 1.86	1.04	0.92 to 1.18
Chronic lung diseases	1.70	1.53 to 1.89	1.25	1.12 to 1.40
Diabetes	1.32	1.20 to 1.44	1.06	0.96 to 1.17
Hypertension	1.86	1.71 to 2.02	1.01	0.92 to 1.12
Immunosuppression	0.79	0.64 to 0.99	0.84	0.66 to 1.07
Education level				
Primary	Ref			
Secondary	0.98	0.89 to 1.08	1.08	0.97 to 1.19
Tertiary	0.86	0.77 to 0.95	0.98	0.87 to 1.10
Region of birth				
Sweden	Ref			
Africa	0.38	0.26 to 0.56	0.70	0.47 to 1.03
Europe	1.02	0.91 to 1.15	0.94	0.83 to 1.06
The Americas	0.55	0.37 to 0.83	0.79	0.52 to 1.19
Asia and Oceania	0.42	0.33 to 0.54	0.76	0.58 to 0.99
*Care-related factors*				
Surgery before index date	0.67	0.57 to 0.80	0.82	0.68 to 0.99
No. of transfers				
0	Ref			
1	1.47	1.35 to 1.60	1.23	1.04 to 1.45
2+	2.62	2.19 to 3.13	2.48	1.89 to 3.24
Hospital				
Danderyd	1.13	1.02 to 1.26	1.05	0.86 to 1.28
Karolinska Huddinge	0.60	0.53 to 0.68	0.68	0.55 to 0.83
Karolinska Solna	0.48	0.40 to 0.56	0.66	0.53 to 0.83
South General	1.25	1.12 to 1.39	1.02	0.85 to 1.23
St Göran	1.34	1.22 to 1.48	1.14	0.95 to 1.36
Södertälje	0.88	0.72 to 1.09	0.72	0.55 to 0.96
Norrtälje	1.84	1.48 to 2.29	1.30	0.98 to 1.73
Other	1.43	1.32 to 1.55	0.82	0.69 to 0.97
Hospital unit				
Geriatric	3.43	3.16 to 3.73	1.54	1.26 to 1.89
Haematology/Oncology/Transplant	0.43	0.31 to 0.60	0.62	0.42 to 0.90
Infection	0.91	0.73 to 1.14	0.68	0.52 to 0.90
Medicine	1.22	1.12 to 1.33	0.76	0.63 to 0.92
Psychiatric	0.29	0.26 to 0.34	0.45	0.35 to 0.58
Surgery	1.06	0.96 to 1.16	0.77	0.64 to 0.93
Other	0.40	0.34 to 0.48	0.55	0.42 to 0.71

^a^Includes patients with their first recorded SARS-CoV-2 infection

^b^Model adjusted for age groups, sex, comorbidities, education level, region of birth, number of vaccine doses at index date, surgery status before index date, number of transfers, hospital, and hospital unit. Comorbidities included cancer, cardiovascular diseases, chronic kidney diseases, chronic lung diseases, diabetes, hypertension, and immunosuppression modelled as binary (yes/no) variables based on ICD-10 codes (eTable1). Hospitals and hospital units were coded as independent binary variables (yes/no) to account for patients moving between different care facilities during their care episode. Sex and surgery status before index date were modelled as binary variables, and age groups, education level, region of birth, number of vaccine doses and number of transfers as categorical variables

### Care-related factors associated with nosocomial SARS-CoV-2 infections

The distribution of care-related factors among cases and controls (Table 3) showed that cases had lower percentage of 0 transfers in the last 7 days prior to testing positive (51.5%) compared to controls (61.5%) but higher percentage of 1 transfer (42.5% for cases vs. 35.7% for controls). Overall and across the 3 time periods, most cases were admitted to geriatric hospital units (57.8%) followed by medicine hospital units (19.1%). The hospital with most patient admissions in the last 7 days before index date among cases were smaller hospitals grouped as Other at 49.4% followed by St Göran at 12.2%, and Danderyd at 12.1%. A small proportion of cases, 5.7%, had surgery in the last 7 days before their infection (Table 3).

**Table 3:** Care-related factors among cases^a^ and controls assessed in last 7 days prior to index date

	**Overall**		**Pre-Vaccination**		**Period 1**		**Period 2**	
	**Controls (N=27065)**	**Cases (N=2711)**	**Controls (N=7223)**	**Cases (N=724)**	**Controls (N=3321)**	**Cases (N=333)**	**Controls (N=16521)**	**Cases (N=1654)**
Surgery before index date	2219 (8.2%)	154 (5.7%)	583 (8.1%)	41 (5.7%)	274 (8.3%)	17 (5.1%)	1362 (8.2%)	96 (5.8%)
Number of transfers								
0	16636 (61.5%)	1396 (51.5%)	4577 (63.4%)	369 (51.0%)	2162 (65.1%)	198 (59.5%)	9897 (59.9%)	829 (50.1%)
1	9661 (35.7%)	1152 (42.5%)	2463 (34.1%)	306 (42.3%)	1065 (32.1%)	119 (35.7%)	6133 (37.1%)	727 (44.0%)
2+	768 (2.8%)	163 (6.0%)	183 (2.5%)	49 (6.8%)	94 (2.8%)	16 (4.8%)	491 (3.0%)	98 (5.9%)
Hospital								
Danderyd	3225 (11.9%)	328 (12.1%)	782 (10.8%)	77 (10.6%)	406 (12.2%)	35 (10.5%)	2037 (12.3%)	216 (13.1%)
Karolinska Huddinge	3509 (13.0%)	212 (7.8%)	1021 (14.1%)	36 (5.0%)	475 (14.3%)	44 (13.2%)	2013 (12.2%)	132 (8.0%)
Karolinska Solna	1789 (6.6%)	93 (3.4%)	482 (6.7%)	31 (4.3%)	223 (6.7%)	15 (4.5%)	1084 (6.6%)	47 (2.8%)
South General	1959 (7.2%)	226 (8.3%)	500 (6.9%)	47 (6.5%)	234 (7.0%)	39 (11.7%)	1225 (7.4%)	140 (8.5%)
St Göran	3032 (11.2%)	332 (12.2%)	774 (10.7%)	116 (16.0%)	356 (10.7%)	41 (12.3%)	1902 (11.5%)	175 (10.6%)
Södertälje	1007 (3.7%)	92 (3.4%)	236 (3.3%)	38 (5.2%)	133 (4.0%)	4 (1.2%)	638 (3.9%)	50 (3.0%)
Norrtälje	491 (1.8%)	89 (3.3%)	127 (1.8%)	16 (2.2%)	49 (1.5%)	4 (1.2%)	315 (1.9%)	69 (4.2%)
Other hospitals	12053 (44.5%)	1339 (49.4%)	3301 (45.7%)	363 (50.1%)	1445 (43.5%)	151 (45.3%)	7307 (44.2%)	825 (49.9%)
Hospital unit								
Geriatric	8691 (32.1%)	1567 (57.8%)	2188 (30.3%)	433 (59.8%)	1067 (32.1%)	136 (40.8%)	5436 (32.9%)	998 (60.3%)
Haematology/Oncology/Transplant	646 (2.4%)	21 (0.8%)	224 (3.1%)	8 (1.1%)	79 (2.4%)	5 (1.5%)	343 (2.1%)	8 (0.5%)
Infection	671 (2.5%)	53 (2.0%)	163 (2.3%)	8 (1.1%)	104 (3.1%)	14 (4.2%)	404 (2.4%)	31 (1.9%)
Medicine	4648 (17.2%)	517 (19.1%)	1122 (15.5%)	140 (19.3%)	522 (15.7%)	71 (21.3%)	3004 (18.2%)	306 (18.5%)
Psychiatric	6656 (24.6%)	235 (8.7%)	1961 (27.1%)	32 (4.4%)	897 (27.0%)	61 (18.3%)	3798 (23.0%)	142 (8.6%)
Surgery	2628 (9.7%)	212 (7.8%)	704 (9.7%)	65 (9.0%)	312 (9.4%)	23 (6.9%)	1612 (9.8%)	124 (7.5%)
Other units	3125 (11.5%)	106 (3.9%)	861 (11.9%)	38 (5.2%)	340 (10.2%)	23 (6.9%)	1924 (11.6%)	45 (2.7%)

*Abbreviation*: *IQR* Interquartile range

^a^Includes patients with their first recorded SARS-CoV-2 infection

For the care-related factors, the adjusted conditional logistic regression analysis showed that admission to geriatric hospital units (aOR: 1.54, 95% CI: 1.26 to 1.89) and increased number of transfers (1 transfer: aOR: 1.23, 95% CI: 1.04 to 1.45; 2 + transfers, aOR: 2.48, 95% CI: 1.89 to 2.34) were associated with increased odds of acquiring nosocomial SARS-CoV-2 infections (Table 2), which was consistent in supplementary analyses of patients > 65 years of age (eTable2). Admittance to Karolinska Huddinge, Karolinska Solna, Södertälje, Other hospitals, and any hospital unit apart from geriatric hospital units were all associated with lower odds of nosocomial infection.

The analysis restricted to cases with ct values below 30 (eTable3) and analysis excluding controls that later became cases (eTable4) showed comparable results to the main analyses. The analysis stratified by time periods showed higher odds of infection for cardiovascular diseases in period 1 (aOR: 1.53, 95% CI: 1.17 to 2.00) and Norrtälje hospital in period 2 (aOR: 1.54, 95% CI: 1.08 to 2.18) (eTable5).

### Association between vaccination and nosocomial SARS-CoV-2 infections

**Fig. 1:**
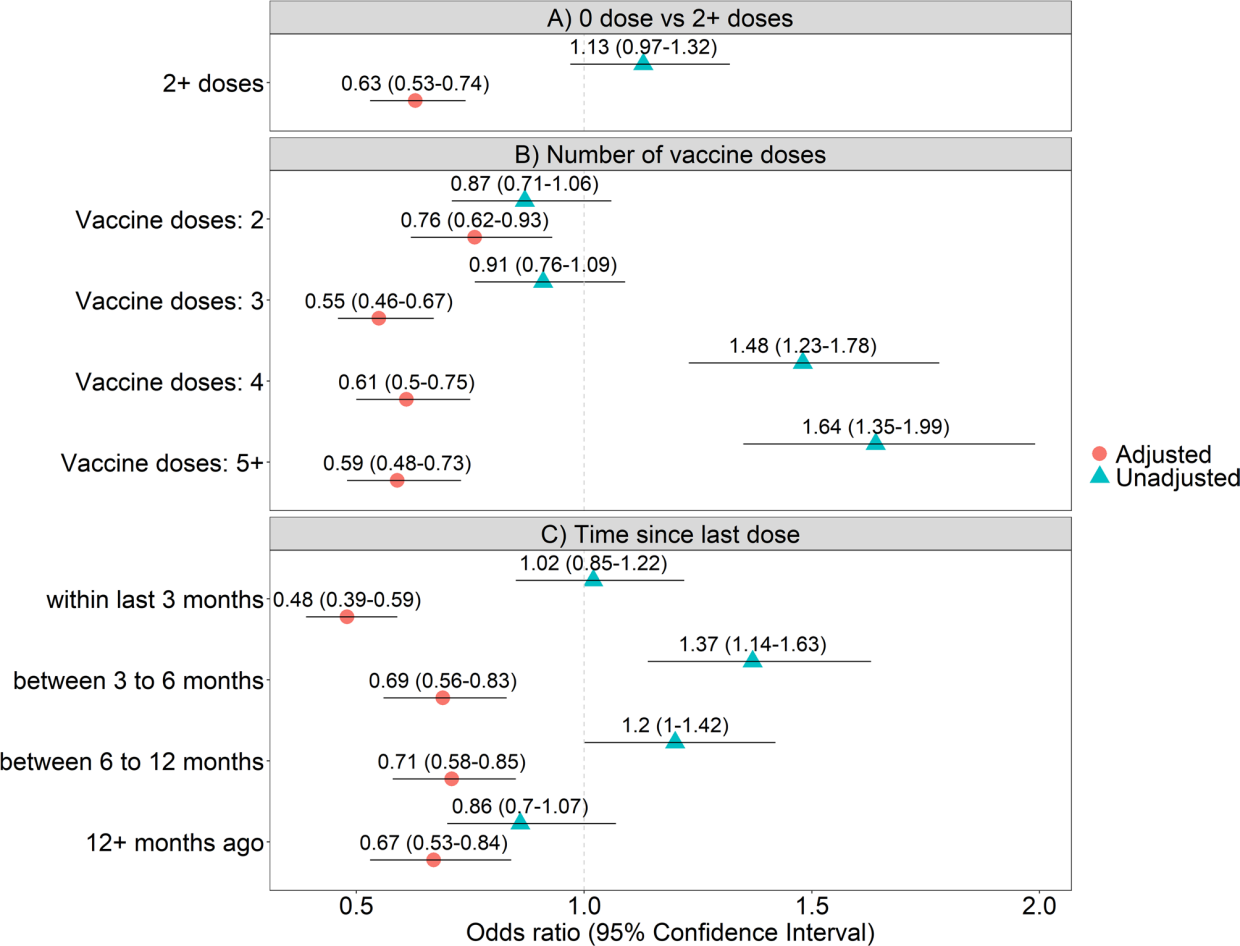
Odds ratio for association between vaccination and nosocomial SARS-CoV-2 infection comparing cases and controls. **A** comparing patients with 0 vaccine doses to those with 2+ vaccine doses. **B** comparing patients with 0 vaccine doses to those with 2, 3, 4, 5+ vaccine doses. **C** comparing patients with 0 vaccine doses to those with 2+ vaccine doses such that the last vaccine dose was administered within last 3 months, between 3 to 6 months, between 6 to 12 months and 12+ months ago. All patients had their vaccine dose counted until 14 days prior to index date to account for development of complete immune response post vaccination. Analyses were restricted to period 1 and 2

While the unadjusted model showed no association between 2 + vaccine doses and nosocomial SARS-CoV-2 infections with odds ratio of 1.13 (95% CI: 0.97 to 1.32), upon adjusting for individual and care-related factors the odds ratio decreased to 0.63 (95% CI: 054 to 0.74) (Fig. 1A). The supplementary analysis with additional matching on hospital and hospital unit showed comparable results (eFigure 1). While patients with one vaccine dose were excluded from the main analysis due to being considered incompletely vaccinated, the sensitivity analysis including this group showed no significant differences compared to the main results. The results showed a non-significant reduced risk suggesting incomplete or limited protection from vaccination with a single dose (eFigure2).

The multivariate analysis on the number of vaccine doses showed that compared to those with 0 doses, vaccination with 3, 4, and 5 + doses were associated with lower odds of infection (Fig. 1B). The analysis on time since last vaccine dose and risk of infection showed that those with last vaccine dose administered within last 3 months had lowest odds of infection (aOR: 0.48, 95% CI: 0.39 to 0.59) (Fig. 1C).

## Discussion

In this population-based study we show that the risk of infection was highest among the elderly and patients admitted to geriatric hospital units, which indicates a need to better implement infection prevention measures to protect a vulnerable group. Additionally, based on the available literature, this is the first study assessing the association between patient vaccination status and risk of nosocomial SARS-CoV-2 infection acquisition, where notably vaccination was associated with a reduced risk of nosocomial SARS-CoV-2 infections, highlighting the importance of vaccination as an effective preventive measure.

Studies analysing the association between age and risk of nosocomial SARS-CoV-2 infections have shown mixed results; a study from the UK identified significant difference in age when comparing nosocomial vs. community-acquired SARS-CoV-2 infected patients [19], whereas a study from France found significant effect of age in bivariate analysis but not in multivariate analysis [7]. However, these studies compared patients with community-acquired SARS-CoV-2 to those with nosocomial SARS-CoV-2, precluding comparisons assessing the risk between age and nosocomial SARS-CoV-2 infection, given the two groups have different demographics and environments when assessing risk for infection. Moreover, in studies reporting no risk of age on developing nosocomial SARS-CoV-2 infection, smaller sample sizes have been used; however, the baseline characteristics showed that the infection occurred among hospitalised patients with a median age 70 years or above which are in line with our results [5, 6].

While in this study, men had a higher risk of developing nosocomial SARS-CoV-2 infections, other studies have shown mixed results. A study from Germany found no differences in risk between men and women when analysing risk factors for nosocomial SARS-CoV-2 [6], whereas a study from Brazil analysing the incidence of nosocomial infections identified most cases among men but lacked adjusted analyses [20]. However, a study using data from the primary care network in the UK and a meta-analysis on risk for COVID-19 infection showed that men have a higher risk for SARS-CoV-2 infection and test positivity [21, 22]. We observed that chronic lung diseases were significantly associated with increased risk of infection. As shown by previous studies, patient with pre-existing respiratory diseases have shown to have increased susceptibility to SARS-CoV-2 infection [23].

Among the care-related factors, increased number of transfers during a care episode led to increased risk of infection. A study from the UK analysing nosocomial SARS-CoV-2 outbreak showed that patient movement through ward transfers led to significant risk of outbreaks, which could be attributable to patients being exposed more frequently to infectious contacts [24]. This was also demonstrated in another study from the UK which analysed transmission of nosocomial COVID-19 on a stroke ward [25]. Admission to geriatric hospital units was significantly associated with increased risk of infection attributable to the increased vulnerability due to comorbidities and frailty [26]. Similar results were observed among outbreak clusters in a hospital in France and among 3 hospitals in a Scottish health board [27, 28].

Our study showed that admittance to certain hospitals was associated with lower risk of infection. Structural factors of the hospitals could have played a role in reducing the risk of infection acquisition such that Karolinska Solna primarily has single-patient rooms compared to other hospitals with several beds in each room [29]. Studies have shown that patients in multi-bedded rooms had increased risk of nosocomial infections which could be attributable to increased exposure [5]. A study on changes to emergency department structure and workflow at Karolinska Huddinge during the early phase of the pandemic showed a decrease in crowding and reduced bed occupancy [30] which highlights that differences in implementation of infection prevention measures between hospitals in the same region can influence risk of infection.

The odds of reduced risk of nosocomial SARS-CoV-2 infection among patients vaccinated with 2 + doses amount to a protective vaccine effectiveness of 26–47% which increased to 41–61% effectiveness if vaccination was within the last 3 months. These findings are consistent with studies assessing vaccine effectiveness in the general population [31, 32] Also, studies have previously analysed the association between vaccination and SARS-CoV-2 infection among HCWs which showed lower infection rates after vaccination [33–35]. Previous studies have primarily assessed the incidence of nosocomial SARS-CoV-2 infections during the vaccination period but not established the association between vaccine doses or time since last vaccine on risk of infection [9, 36].

There are limitations to this study. Due to lack of availability of data, we could not assess care-related factors such as ventilation in hospitals, bed occupancy, single or multi-bedded rooms, which have been previously studied [2, 5, 6, 37]. However, by adjusting for the hospital and hospital unit, we can use the institution as a proxy for these measures. We lacked data on antigen tests performed at home or outside of hospitals, which might have led to some misclassification of previous infections. Also, we lacked information on the indication to perform SARS-CoV-2 test in hospitalised patients as being part of screening procedures or upon symptoms onset. In this study, contact patterns between patients and healthcare providers are not assessed, which has previously been investigated [2, 37]. We excluded patients with a main diagnosis of COVID-19 at admission to focus on patients at risk of acquiring nosocomial SARS-CoV-2 during their hospitalisation and excluded patients with LOS > 90 days which could have underestimated the burden of infection. Finally, the case definition of nosocomial SARS-CoV-2 infections varies between studies, and while we used the ECDC definition for classification of infections [10] and large-scale register data instead of clinical records, there could be misclassification of community-acquired infections as nosocomial given long incubation period or nosocomial cases as community-acquired or indeterminate given short incubation period biasing the results in either direction. We lacked detailed information on descriptors related to transfers during a care episode; however, the findings highlight an important and actionable risk factor that is applicable across healthcare settings. Strengths of the study are that we used a large study population with complete data on healthcare admissions, patient socio-demographic characteristics, PCR testing data and vaccination status, from all hospitals in Region Stockholm, thereby increasing the internal and external validity.

## Conclusions

This study identifies key risk factors associated with increased risk of nosocomial SARS-CoV-2 infections, where individual-related factors assist in identifying high-risk patient groups, and care-related factors points to high-risk areas where transmission could be reduced through strategic planning of care services and implementation of infection prevention measures. The protective effect of vaccination against infection for nosocomial SARS-CoV-2 helps provide evidence for clinicians and policy makers on the protective effect of vaccination in reducing nosocomial SARS-CoV-2 infections.

## Supplementary Information

Supplementary Material 1.

## Data Availability

The data on individual participants used in this study were obtained using ethical approval and therefore it cannot be shared publicly. Due to protection of personal integrity of participants, data from the deidentified administrative health registry are not freely available.

## Abbreviations

SARS-CoV-2: Severe acute respiratory syndrome coronavirus 2
LOS: Length of stay
HCWs: Healthcare workers
PCR: Polymerase chain reaction
CT: Cycle threshold
VAL: Region Stockholm Healthcare Data Warehouse
IQR: Interquartile range
