# Supplementary material for: Risk factors and effectiveness of vaccination for nosocomial SARS-CoV-2 acquisition throughout the SARS-CoV-2 pandemic
Source: BMC Infect Dis. 2025 Aug 19;25:1040. doi: 10.1186/s12879-025-11349-9 (PMC12366111; doi:10.1186/s12879-025-11349-9)
Supplement: Supplementary file 1 — Supplementary Material 1. [file 12879_2025_11349_MOESM1_ESM.docx]

**eSupplement**

**Title: Risk factors and effectiveness of vaccination for nosocomial SARS-CoV-2 acquisition throughout the SARS-CoV-2 pandemic**

**Table of Contents**

- eTable1: ICD-10 codes for comorbidities
- eTable2: Unadjusted and adjusted odds ratio for analysis restricted to patients aged 65 years and older
- eTable3: Unadjusted and adjusted odds ratio for risk factors of nosocomial SARS-CoV-2 including cases with cycle threshold (ct) values ≤ 30 and matched controls
- eTable4: Unadjusted and adjusted odds ratio for risk factors of nosocomial SARS-CoV-2 excluding cases which acted as controls
- eTable5: Unadjusted and adjusted odds ratio for analysis stratified by time periods
- eFigure1: Forest plot showing the unadjusted and adjusted odds ratio for vaccine effectiveness comparing patients with 0 vaccine doses to 2+ vaccine doses in updated matched dataset
- eFigure2: Odds ratio for association between vaccination and nosocomial SARS-CoV-2 infection comparing cases and controls including patients with 1 vaccine dose

# eTable1. ICD-10 codes for comorbidities

| **Comorbidity category** | **ICD-10 codes^a^** |
| --- | --- |
| Cancer | C0-C1, C4-C7, C20-C26, C30-C34, C37-C39, C80-C96 |
| Chronic kidney disease | N18 |
| Chronic lung diseases | I26, I27.0, I27.2, J47, J70.2-J70.4, J84, J98.2, J99.0, M05.1 |
| Cardiac diseases | I05-I08, I20-I22, I24-I28, I34-I37, I42, I44-I50 |
| Diabetes | E10-E14 |
| Hypertension | I10-I15 |
| Immunosuppression | D70-D72, D73.0, D80-D84, Z51.0, Z51.1 |

**^a^** Comorbidities included in the study are based on ICD-10 codes recorded up to 3 years before the start of study.

# eTable2. Unadjusted and adjusted^a^ odds ratio for analysis restricted to patients aged 65 years and older

|  | Unadjusted | | Adjusted | |
| --- | --- | --- | --- | --- |
| Variable | OR | 95% CI | OR | 95% CI |
| *Individual-related factors* | | | | |
| Age groups (in years) | | | | |
| 61-70 | Ref | | | |
| 71-80 | 1.54 | 1.30 to 1.82 | 1.19 | 1.00 to 1.41 |
| 81-90 | 2.39 | 2.03 to 2.82 | 1.45 | 1.21 to 1.73 |
| 90+ | 3.21 | 2.67 to 3.87 | 1.74 | 1.42 to 2.14 |
| Sex | | | | |
| Women | Ref | | | |
| Men | 1.02 | 0.94 to 1.12 | 1.15 | 1.05 to 1.26 |
| Comorbidities |  |  |  |  |
| Cancer | 0.99 | 0.89 to 1.10 | 0.95 | 0.85 to 1.07 |
| Cardiovascular diseases | 1.37 | 1.25 to 1.49 | 1.07 | 0.97 to 1.18 |
| Chronic kidney diseases | 1.31 | 1.16 to 1.47 | 1.03 | 0.91 to 1.17 |
| Chronic lung diseases | 1.33 | 1.19 to 1.48 | 1.25 | 1.12 to 1.41 |
| Diabetes | 1.06 | 0.96 to 1.17 | 1.03 | 0.93 to 1.15 |
| Hypertension | 1.23 | 1.12 to 1.35 | 1.00 | 0.90 to 1.11 |
| Immunosuppression | 0.70 | 0.55 to 0.88 | 0.85 | 0.65 to 1.09 |
| Education level | | | | |
| Primary | Ref | | | |
| Secondary | 0.96 | 0.86 to 1.07 | 1.06 | 0.95 to 1.18 |
| Tertiary | 0.81 | 0.72 to 0.91 | 0.95 | 0.84 to 1.07 |
| Region of birth | | | | |
| Sweden | Ref | | | |
| Africa | 0.35 | 0.18 to 0.66 | 0.49 | 0.26 to 0.93 |
| Europe | 0.96 | 0.85 to 1.09 | 0.97 | 0.85 to 1.10 |
| The Americas | 0.58 | 0.35 to 0.94 | 0.71 | 0.43 to 1.16 |
| Asia and Oceania | 0.60 | 0.43 to 0.83 | 0.84 | 0.60 to 1.19 |
| *Care-related factors* | | | | |
| Surgery before index date | 0.62 | 0.51 to 0.74 | 0.80 | 0.66 to 0.99 |
| Number of transfers | | | | |
| 0 | Ref | | | |
| 1 | 1.13 | 1.03 to 1.23 | 1.15 | 0.95 to 1.39 |
| 2+ | 2.12 | 1.75 to 2.57 | 2.39 | 1.78 to 3.21 |
| Hospital | | | | |
| Danderyd | 1.16 | 1.04 to 1.30 | 1.08 | 0.87 to 1.34 |
| Karolinska Huddinge | 0.64 | 0.56 to 0.74 | 0.69 | 0.55 to 0.87 |
| Karolinska Solna | 0.44 | 0.36 to 0.53 | 0.66 | 0.51 to 0.85 |
| South General | 1.03 | 0.92 to 1.16 | 1.02 | 0.84 to 1.25 |
| St Göran | 1.28 | 1.15 to 1.42 | 1.10 | 0.91 to 1.34 |
| Södertälje | 0.94 | 0.76 to 1.17 | 0.75 | 0.55 to 1.02 |
| Norrtälje | 1.51 | 1.20 to 1.90 | 1.32 | 0.97 to 1.78 |
| Other | 1.30 | 1.19 to 1.42 | 0.86 | 0.71 to 1.03 |
| Hospital unit | | | | |
| Geriatric | 2.54 | 2.31 to 2.80 | 1.55 | 1.24 to 1.94 |
| Haematology/Oncology/Transplant | 0.41 | 0.27 to 0.61 | 0.64 | 0.41 to 1.01 |
| Infection | 0.83 | 0.66 to 1.05 | 0.73 | 0.54 to 0.98 |
| Medicine | 0.95 | 0.87 to 1.04 | 0.77 | 0.63 to 0.95 |
| Psychiatric | 0.43 | 0.35 to 0.53 | 0.51 | 0.38 to 0.69 |
| Surgery | 0.93 | 0.84 to 1.03 | 0.82 | 0.67 to 1.01 |
| Other | 0.31 | 0.25 to 0.39 | 0.50 | 0.37 to 0.68 |

^a^ Model adjusted for age groups, sex, comorbidities, education level, region of birth, number of vaccine doses at index date, surgery status before index date, number of transfers, hospital, and hospital unit. Comorbidities included cancer, cardiovascular diseases, chronic kidney diseases, chronic lung diseases, diabetes, hypertension, and immunosuppression modelled as binary (yes/no) variables based on ICD-10 codes (eTable 1 in supplement). Hospitals and hospital units were coded as independent binary variables (yes/no) to account for patients moving between different care facilities during their care episode. Sex and surgery status before index date were modelled as binary variables, and age groups, education level, region of birth, number of vaccine doses and number of transfers as categorical variables.

# eTable3: Unadjusted and adjusted^a^ odds ratio for risk factors of nosocomial SARS-CoV-2 including cases with cycle threshold (ct) values ≤ 30 and matched controls

|  | Unadjusted | | Adjusted | |
| --- | --- | --- | --- | --- |
|  | **OR** | **95% CI** | **OR** | **95% CI** |
| *Individual-related factors* | | | | |
| Age groups (in years) |  | | | |
| 18-30 | Ref | | | |
| 31-40 | 1.82 | 0.74 to 4.49 | 1.79 | 0.72 to 4.44 |
| 41-50 | 2.63 | 1.13 to 6.11 | 2.60 | 1.11 to 6.10 |
| 51-60 | 4.02 | 1.83 to 8.84 | 3.52 | 1.58 to 7.81 |
| 61-70 | 5.70 | 2.65 to 12.28 | 3.90 | 1.77 to 8.58 |
| 71-80 | 10.30 | 4.85 to 21.87 | 5.25 | 2.40 to 11.49 |
| 81-90 | 15.24 | 7.18 to 32.35 | 6.16 | 2.80 to 13.57 |
| 90+ | 23.07 | 10.77 to 49.43 | 8.29 | 3.72 to 18.48 |
| Sex |  |  |  |  |
| Women | Ref | | | |
| Men | 1.04 | 0.93 to 1.17 | 1.18 | 1.04 to 1.33 |
| Comorbidities |  |  |  |  |
| Cancer | 1.29 | 1.12 to 1.50 | 0.99 | 0.84 to 1.16 |
| Cardiovascular diseases | 1.91 | 1.70 to 2.14 | 1.08 | 0.94 to 1.23 |
| Chronic kidney diseases | 1.58 | 1.34 to 1.86 | 0.95 | 0.80 to 1.13 |
| Chronic lung diseases | 1.74 | 1.50 to 2.02 | 1.27 | 1.09 to 1.49 |
| Diabetes | 1.35 | 1.18 to 1.53 | 1.07 | 0.93 to 1.24 |
| Hypertension | 1.85 | 1.64 to 2.09 | 0.98 | 0.85 to 1.13 |
| Immunosuppression | 0.70 | 0.51 to 0.97 | 0.74 | 0.52 to 1.06 |
| Education level |  |  |  |  |
| Primary | Ref | | | |
| Secondary | 0.92 | 0.80 to 1.06 | 1.02 | 0.88 to 1.18 |
| Tertiary | 0.82 | 0.71 to 0.95 | 0.95 | 0.81 to 1.12 |
| Region of birth |  |  |  |  |
| Sweden | Ref | | | |
| Africa | 0.42 | 0.25 to 0.70 | 0.89 | 0.52 to 1.52 |
| Europe | 1.05 | 0.89 to 1.23 | 0.99 | 0.83 to 1.18 |
| The Americas | 0.67 | 0.39 to 1.13 | 0.95 | 0.55 to 1.64 |
| Asia and Oceania | 0.54 | 0.38 to 0.76 | 1.01 | 0.70 to 1.45 |
| *Care-related factors* | | | | |
| Surgery before index date | 0.65 | 0.51 to 0.84 | 0.85 | 0.65 to 1.12 |
| Number of transfers |  | | | |
| 0 | Ref | | | |
| 1 | 1.50 | 1.33 to 1.70 | 1.31 | 1.03 to 1.67 |
| 2+ | 2.88 | 2.25 to 3.70 | 3.13 | 2.13 to 4.60 |
| Hospital |  |  |  |  |
| Danderyd | 1.34 | 1.16 to 1.55 | 1.17 | 0.89 to 1.55 |
| Karolinska Huddinge | 0.58 | 0.48 to 0.70 | 0.62 | 0.46 to 0.84 |
| Karolinska Solna | 0.46 | 0.36 to 0.58 | 0.65 | 0.47 to 0.90 |
| South General | 1.38 | 1.19 to 1.60 | 1.09 | 0.84 to 1.43 |
| St Göran | 1.06 | 0.91 to 1.23 | 0.87 | 0.67 to 1.13 |
| Södertälje | 0.51 | 0.35 to 0.74 | 0.40 | 0.25 to 0.63 |
| Norrtälje | 1.94 | 1.44 to 2.61 | 1.30 | 0.87 to 1.94 |
| Other | 1.55 | 1.38 to 1.74 | 0.82 | 0.64 to 1.05 |
| Hospital unit |  |  |  |  |
| Geriatric | 3.70 | 3.28 to 4.18 | 1.60 | 1.19 to 2.14 |
| Haematology/Oncology/Transplant | 0.36 | 0.22 to 0.59 | 0.48 | 0.27 to 0.86 |
| Infection | 1.19 | 0.89 to 1.58 | 0.74 | 0.51 to 1.07 |
| Medicine | 1.18 | 1.05 to 1.33 | 0.72 | 0.55 to 0.94 |
| Psychiatric | 0.25 | 0.20 to 0.31 | 0.43 | 0.30 to 0.62 |
| Surgery | 1.05 | 0.91 to 1.20 | 0.69 | 0.53 to 0.91 |
| Other | 0.46 | 0.36 to 0.58 | 0.58 | 0.40 to 0.85 |

^a^ Model adjusted for age groups, sex, comorbidities, education level, region of birth, number of vaccine doses at index date, surgery status before index date, number of transfers, hospital, and hospital unit. Comorbidities included cancer, cardiovascular diseases, chronic kidney diseases, chronic lung diseases, diabetes, hypertension, and immunosuppression modelled as binary (yes/no) variables based on ICD-10 codes (eTable 1 in supplement). Hospitals and hospital units were coded as independent binary variables (yes/no) to account for patients moving between different care facilities during their care episode. Sex and surgery status before index date were modelled as binary variables, and age groups, education level, region of birth, number of vaccine doses and number of transfers as categorical variables.

# eTable4: Unadjusted and adjusted^a^ odds ratio for risk factors of nosocomial SARS-CoV-2 excluding cases which acted as controls

|  | Unadjusted | | Adjusted | |
| --- | --- | --- | --- | --- |
| Variable | OR | 95% CI | OR | 95% CI |
| *Individual-related factors* | | | | |
| Age groups (in years) | | | | |
| 18-30 | Ref | | | |
| 31-40 | 1.38 | 0.87 to 2.19 | 1.36 | 0.86 to 2.17 |
| 41-50 | 1.67 | 1.08 to 2.59 | 1.68 | 1.08 to 2.62 |
| 51-60 | 2.00 | 1.33 to 3.00 | 1.84 | 1.21 to 2.79 |
| 61-70 | 3.15 | 2.15 to 4.62 | 2.30 | 1.54 to 3.43 |
| 71-80 | 5.43 | 3.75 to 7.87 | 2.91 | 1.95 to 4.34 |
| 81-90 | 8.68 | 6.00 to 12.55 | 3.64 | 2.43 to 5.45 |
| 90+ | 11.79 | 8.07 to 17.22 | 4.46 | 2.94 to 6.77 |
| Sex | | | | |
| Women | Ref | | | |
| Men | 0.97 | 0.90 to 1.05 | 1.11 | 1.02 to 1.21 |
| Comorbidities |  |  |  |  |
| Cancer | 1.27 | 1.15 to 1.41 | 0.94 | 0.83 to 1.05 |
| Cardiovascular diseases | 1.93 | 1.78 to 2.09 | 1.08 | 0.99 to 1.19 |
| Chronic kidney diseases | 1.70 | 1.52 to 1.91 | 1.05 | 0.93 to 1.19 |
| Chronic lung diseases | 1.76 | 1.58 to 1.95 | 1.28 | 1.15 to 1.43 |
| Diabetes | 1.34 | 1.22 to 1.47 | 1.08 | 0.97 to 1.19 |
| Hypertension | 1.91 | 1.75 to 2.07 | 1.00 | 0.91 to 1.11 |
| Immunosuppression | 0.79 | 0.63 to 0.98 | 0.84 | 0.66 to 1.07 |
| Education level | | | | |
| Primary | Ref | | | |
| Secondary | 0.99 | 0.90 to 1.10 | 1.10 | 0.99 to 1.21 |
| Tertiary | 0.85 | 0.77 to 0.95 | 0.98 | 0.88 to 1.10 |
| Region of birth | | | | |
| Sweden | Ref | | | |
| Africa | 0.37 | 0.25 to 0.54 | 0.68 | 0.46 to 1.01 |
| Europe | 1.02 | 0.91 to 1.14 | 0.93 | 0.82 to 1.05 |
| The Americas | 0.53 | 0.35 to 0.80 | 0.77 | 0.51 to 1.16 |
| Asia and Oceania | 0.40 | 0.31 to 0.52 | 0.74 | 0.57 to 0.96 |
| *Care-related factors* | | | | |
| Surgery before index date | 0.67 | 0.56 to 0.79 | 0.82 | 0.68 to 0.99 |
| Number of transfers | | | | |
| 0 | Ref | | | |
| 1 | 1.49 | 1.37 to 1.62 | 1.25 | 1.06 to 1.48 |
| 2+ | 2.70 | 2.26 to 3.24 | 2.61 | 1.99 to 3.42 |
| Hospital | | | | |
| Danderyd | 1.14 | 1.02 to 1.26 | 1.06 | 0.87 to 1.29 |
| Karolinska Huddinge | 0.59 | 0.52 to 0.67 | 0.68 | 0.56 to 0.84 |
| Karolinska Solna | 0.46 | 0.39 to 0.54 | 0.66 | 0.53 to 0.82 |
| South General | 1.29 | 1.16 to 1.43 | 1.07 | 0.88 to 1.29 |
| St Göran | 1.37 | 1.24 to 1.51 | 1.16 | 0.97 to 1.39 |
| Södertälje | 0.87 | 0.71 to 1.07 | 0.72 | 0.54 to 0.96 |
| Norrtälje | 1.91 | 1.53 to 2.38 | 1.35 | 1.01 to 1.80 |
| Other | 1.43 | 1.32 to 1.55 | 0.82 | 0.69 to 0.97 |
| Hospital unit |  |  |  |  |
| Geriatric | 3.56 | 3.27 to 3.87 | 1.51 | 1.23 to 1.85 |
| Haematology/Oncology/Transplant | 0.41 | 0.30 to 0.57 | 0.59 | 0.40 to 0.87 |
| Infection | 0.92 | 0.74 to 1.14 | 0.67 | 0.51 to 0.88 |
| Medicine | 1.24 | 1.14 to 1.35 | 0.75 | 0.62 to 0.90 |
| Psychiatric | 0.29 | 0.25 to 0.33 | 0.45 | 0.35 to 0.57 |
| Surgery | 1.06 | 0.96 to 1.17 | 0.76 | 0.63 to 0.92 |
| Other | 0.39 | 0.33 to 0.46 | 0.52 | 0.40 to 0.68 |

^a^ Model adjusted for age groups, sex, comorbidities, education level, region of birth, number of vaccine doses at index date, surgery status before index date, number of transfers, hospital, and hospital unit. Comorbidities included cancer, cardiovascular diseases, chronic kidney diseases, chronic lung diseases, diabetes, hypertension, and immunosuppression modelled as binary (yes/no) variables based on ICD-10 codes (eTable 1 in supplement). Hospitals and hospital units were coded as independent binary variables (yes/no) to account for patients moving between different care facilities during their care episode. Sex and surgery status before index date were modelled as binary variables, and age groups, education level, region of birth, number of vaccine doses and number of transfers as categorical variables.

# eTable5: Unadjusted and adjusted^a^ odds ratio for analysis stratified by time periods

|  | Pre-Vaccination | | | | | | Period 1 | | | | | Period 2 | | | | | |
| --- | --- | --- | --- | --- | --- | --- | --- | --- | --- | --- | --- | --- | --- | --- | --- | --- | --- |
|  | Unadjusted | | | Adjusted | | | Unadjusted | | | Adjusted | | Unadjusted | | | Adjusted | | |
|  | OR | 95% CI | OR | | 95% CI | OR | | 95% CI | OR | | 95% CI | | OR | 95% CI | | OR | 95% CI |
| *Individual-related factors* | | | | | | | | | | | | | | | | | |
| Age groups (in years) |  |  |  | |  |  | |  |  | |  | |  |  | |  |  |
| 18-30 | Ref | | | | | | | | | | | | | | | | |
| 31-40 | 1.77 | 0.65 to 4.84 | 1.63 | | 0.59 to 4.49 | 1.62 | | 0.66 to 4.00 | 1.91 | | 0.77 to 4.77 | | 1.13 | 0.59 to 2.16 | | 1.11 | 0.58 to 2.12 |
| 41-50 | 1.63 | 0.61 to 4.37 | 1.29 | | 0.47 to 3.53 | 1.78 | | 0.76 to 4.20 | 2.07 | | 0.86 to 5.02 | | 1.56 | 0.86 to 2.84 | | 1.60 | 0.87 to 2.92 |
| 51-60 | 2.58 | 1.05 to 6.30 | 1.64 | | 0.65 to 4.12 | 1.53 | | 0.67 to 3.53 | 1.64 | | 0.70 to 3.87 | | 1.96 | 1.13 to 3.41 | | 1.96 | 1.11 to 3.45 |
| 61-70 | 5.72 | 2.48 to 13.20 | 2.71 | | 1.12 to 6.52 | 2.06 | | 0.95 to 4.49 | 1.97 | | 0.86 to 4.52 | | 2.72 | 1.61 to 4.59 | | 2.20 | 1.27 to 3.81 |
| 71-80 | 8.99 | 3.97 to 20.39 | 2.96 | | 1.24 to 7.11 | 2.67 | | 1.27 to 5.61 | 2.39 | | 1.05 to 5.44 | | 5.02 | 3.03 to 8.32 | | 2.82 | 1.63 to 4.86 |
| 81-90 | 15.57 | 6.88 to 35.24 | 3.91 | | 1.62 to 9.46 | 3.33 | | 1.58 to 7.02 | 2.85 | | 1.22 to 6.67 | | 7.81 | 4.72 to 12.93 | | 3.40 | 1.96 to 5.89 |
| 90+ | 21.21 | 9.25 to 48.66 | 4.67 | | 1.89 to 11.50 | 4.18 | | 1.90 to 9.21 | 3.28 | | 1.32 to 8.15 | | 10.49 | 6.26 to 17.57 | | 4.12 | 2.34 to 7.25 |
| Sex |  |  |  | |  |  | |  |  | |  | |  |  | |  |  |
| Women | Ref | | | | | | | | | | | | | | | | |
| Men | 1.03 | 0.89 to 1.20 | 1.14 | | 0.96 to 1.35 | 0.96 | | 0.76 to 1.20 | 0.96 | | 0.76 to 1.23 | | 0.96 | 0.87 to 1.06 | | 1.15 | 1.03 to 1.28 |
| Comorbidities |  |  |  | |  |  | |  |  | |  | |  |  | |  |  |
| Cancer | 1.18 | 0.98 to 1.43 | 0.85 | | 0.68 to 1.06 | 1.13 | | 0.84 to 1.53 | 0.90 | | 0.65 to 1.26 | | 1.34 | 1.17 to 1.53 | | 1.01 | 0.87 to 1.17 |
| Cardiovascular diseases | 2.37 | 2.03 to 2.77 | 1.13 | | 0.94 to 1.36 | 1.83 | | 1.45 to 2.30 | 1.53 | | 1.17 to 2.00 | | 1.68 | 1.52 to 1.87 | | 0.98 | 0.87 to 1.10 |
| Chronic kidney diseases | 2.01 | 1.65 to 2.44 | 1.13 | | 0.91 to 1.41 | 1.23 | | 0.87 to 1.73 | 0.90 | | 0.62 to 1.30 | | 1.59 | 1.36 to 1.85 | | 1.02 | 0.86 to 1.20 |
| Chronic lung diseases | 2.19 | 1.82 to 2.63 | 1.50 | | 1.23 to 1.83 | 1.06 | | 0.76 to 1.47 | 0.80 | | 0.57 to 1.14 | | 1.64 | 1.43 to 1.88 | | 1.21 | 1.05 to 1.40 |
| Diabetes | 1.56 | 1.31 to 1.85 | 1.18 | | 0.97 to 1.42 | 1.14 | | 0.86 to 1.50 | 0.99 | | 0.73 to 1.33 | | 1.25 | 1.11 to 1.41 | | 1.03 | 0.90 to 1.17 |
| Hypertension | 1.94 | 1.66 to 2.28 | 0.85 | | 0.70 to 1.03 | 1.59 | | 1.26 to 2.01 | 1.14 | | 0.86 to 1.51 | | 1.89 | 1.70 to 2.10 | | 1.07 | 0.94 to 1.21 |
| Immunosuppression | 0.76 | 0.54 to 1.07 | 0.91 | | 0.61 to 1.37 | 0.95 | | 0.52 to 1.74 | 1.06 | | 0.55 to 2.03 | | 0.79 | 0.57 to 1.08 | | 0.77 | 0.55 to 1.09 |
| Education level |  |  |  | |  |  | |  |  | |  | |  |  | |  |  |
| Primary | Ref | | | | | | | | | | | | | | | | |
| Secondary | 0.85 | 0.71 to 1.02 | 1.02 | | 0.84 to 1.25 | 1.08 | | 0.82 to 1.42 | 1.14 | | 0.85 to 1.52 | | 1.03 | 0.91 to 1.17 | | 1.11 | 0.97 to 1.26 |
| Tertiary | 0.78 | 0.64 to 0.95 | 0.99 | | 0.80 to 1.24 | 0.97 | | 0.71 to 1.32 | 0.98 | | 0.71 to 1.35 | | 0.87 | 0.76 to 1.00 | | 0.99 | 0.86 to 1.15 |
| Region of birth |  |  |  | |  |  | |  |  | |  | |  |  | |  |  |
| Sweden | Ref | | | | | | | | | | | | | | | | |
| Africa | 0.48 | 0.25 to 0.92 | 1.12 | | 0.57 to 2.22 | 0.70 | | 0.32 to 1.54 | 0.83 | | 0.37 to 1.87 | | 0.26 | 0.14 to 0.47 | | 0.45 | 0.25 to 0.84 |
| Europe | 1.24 | 1.00 to 1.54 | 1.22 | | 0.97 to 1.53 | 0.90 | | 0.65 to 1.26 | 0.83 | | 0.59 to 1.16 | | 0.96 | 0.82 to 1.12 | | 0.87 | 0.75 to 1.02 |
| The Americas | 0.67 | 0.33 to 1.38 | 1.17 | | 0.55 to 2.46 | 0.23 | | 0.03 to 1.65 | 0.33 | | 0.04 to 2.41 | | 0.55 | 0.33 to 0.91 | | 0.76 | 0.45 to 1.28 |
| Asia and Oceania | 0.54 | 0.34 to 0.86 | 1.21 | | 0.74 to 1.98 | 0.57 | | 0.30 to 1.09 | 0.72 | | 0.37 to 1.41 | | 0.35 | 0.24 to 0.49 | | 0.63 | 0.44 to 0.91 |
| *Care-related factors* | | | | | | | | | | | | | | | | | |
| Surgery before index date | 0.68 | 0.49 to 0.95 | 0.81 | | 0.56 to 1.17 | 0.60 | | 0.36 to 0.99 | 0.59 | | 0.34 to 1.02 | | 0.68 | 0.55 to 0.85 | | 0.88 | 0.69 to 1.11 |
| Number of transfers |  |  |  | |  |  | |  |  | |  | |  |  | |  |  |
| 0 | Ref | | | | | | | | | | | | | | | | |
| 1 | 1.61 | 1.37 to 1.90 | 1.13 | | 0.80 to 1.59 | 1.26 | | 0.98 to 1.61 | 1.35 | | 0.85 to 2.15 | | 1.45 | 1.30 to 1.62 | | 1.19 | 0.96 to 1.48 |
| 2+ | 3.54 | 2.52 to 4.98 | 2.98 | | 1.72 to 5.18 | 1.91 | | 1.10 to 3.32 | 2.71 | | 1.25 to 5.87 | | 2.44 | 1.94 to 3.07 | | 2.20 | 1.56 to 3.10 |
| Hospital |  |  |  | |  |  | |  |  | |  | |  |  | |  |  |
| Danderyd | 1.15 | 0.94 to 1.42 | 1.02 | | 0.69 to 1.52 | 0.77 | | 0.55 to 1.08 | 0.59 | | 0.32 to 1.09 | | 1.20 | 1.06 to 1.36 | | 1.16 | 0.91 to 1.49 |
| Karolinska Huddinge | 0.37 | 0.28 to 0.49 | 0.42 | | 0.27 to 0.65 | 0.84 | | 0.61 to 1.15 | 0.74 | | 0.42 to 1.31 | | 0.66 | 0.56 to 0.77 | | 0.78 | 0.60 to 1.01 |
| Karolinska Solna | 0.52 | 0.39 to 0.70 | 0.67 | | 0.43 to 1.02 | 0.75 | | 0.50 to 1.12 | 0.71 | | 0.39 to 1.31 | | 0.41 | 0.33 to 0.51 | | 0.63 | 0.47 to 0.85 |
| South General | 1.02 | 0.82 to 1.28 | 0.80 | | 0.55 to 1.17 | 1.62 | | 1.22 to 2.15 | 1.09 | | 0.64 to 1.87 | | 1.27 | 1.11 to 1.45 | | 1.10 | 0.87 to 1.39 |
| St Göran | 1.90 | 1.60 to 2.26 | 1.44 | | 1.02 to 2.03 | 1.26 | | 0.93 to 1.69 | 0.96 | | 0.56 to 1.65 | | 1.15 | 1.01 to 1.31 | | 1.03 | 0.82 to 1.30 |
| Södertälje | 1.55 | 1.10 to 2.19 | 1.12 | | 0.66 to 1.91 | 0.26 | | 0.10 to 0.71 | 0.22 | | 0.07 to 0.69 | | 0.78 | 0.60 to 1.02 | | 0.68 | 0.47 to 0.98 |
| Norrtälje | 1.62 | 1.03 to 2.56 | 1.08 | | 0.60 to 1.94 | 0.70 | | 0.25 to 1.94 | 0.40 | | 0.13 to 1.27 | | 2.12 | 1.63 to 2.75 | | 1.54 | 1.08 to 2.18 |
| Other | 1.42 | 1.22 to 1.66 | 0.63 | | 0.45 to 0.89 | 1.28 | | 1.02 to 1.61 | 0.80 | | 0.47 to 1.37 | | 1.46 | 1.32 to 1.62 | | 0.89 | 0.72 to 1.10 |
| Hospital unit |  |  |  | |  |  | |  |  | |  | |  |  | |  |  |
| Geriatric | 4.40 | 3.73 to 5.19 | 1.98 | | 1.30 to 3.01 | 1.52 | | 1.21 to 1.92 | 0.85 | | 0.48 to 1.52 | | 3.66 | 3.28 to 4.07 | | 1.63 | 1.26 to 2.11 |
| Haematology/Oncology/Transplant | 0.36 | 0.19 to 0.68 | 0.66 | | 0.31 to 1.42 | 0.63 | | 0.29 to 1.37 | 0.71 | | 0.28 to 1.84 | | 0.42 | 0.27 to 0.66 | | 0.60 | 0.36 to 1.00 |
| Infection | 0.65 | 0.39 to 1.09 | 0.58 | | 0.31 to 1.09 | 1.24 | | 0.75 to 2.03 | 0.96 | | 0.49 to 1.90 | | 0.94 | 0.71 to 1.24 | | 0.66 | 0.46 to 0.94 |
| Medicine | 1.40 | 1.19 to 1.65 | 0.79 | | 0.53 to 1.18 | 1.35 | | 1.06 to 1.72 | 0.92 | | 0.54 to 1.58 | | 1.13 | 1.02 to 1.26 | | 0.76 | 0.60 to 0.96 |
| Psychiatric | 0.15 | 0.10 to 0.20 | 0.27 | | 0.16 to 0.46 | 0.57 | | 0.43 to 0.77 | 0.67 | | 0.35 to 1.28 | | 0.31 | 0.26 to 0.37 | | 0.51 | 0.37 to 0.70 |
| Surgery | 1.20 | 1.00 to 1.43 | 0.87 | | 0.59 to 1.28 | 0.92 | | 0.69 to 1.24 | 0.79 | | 0.45 to 1.37 | | 1.03 | 0.91 to 1.16 | | 0.76 | 0.60 to 0.97 |
| Other | 0.45 | 0.33 to 0.62 | 0.79 | | 0.47 to 1.33 | 0.93 | | 0.64 to 1.35 | 0.81 | | 0.41 to 1.57 | | 0.29 | 0.23 to 0.38 | | 0.42 | 0.29 to 0.60 |

^a^ Model adjusted for age groups, sex, comorbidities, education level, region of birth, number of vaccine doses at index date, surgery status before index date, number of transfers, hospital, and hospital unit. Comorbidities included cancer, cardiovascular diseases, chronic kidney diseases, chronic lung diseases, diabetes, hypertension, and immunosuppression modelled as binary (yes/no) variables based on ICD-10 codes (eTable 1 in supplement). Hospitals and hospital units were coded as independent binary variables (yes/no) to account for patients moving between different care facilities during their care episode. Sex and surgery status before index date were modelled as binary variables, and age groups, education level, region of birth, number of vaccine doses and number of transfers as categorical variables.

# eFigure1: Forest plot showing the unadjusted and adjusted odds ratio for vaccine effectiveness comparing patients with 0 doses to 2+ doses in updated matched dataset^a^


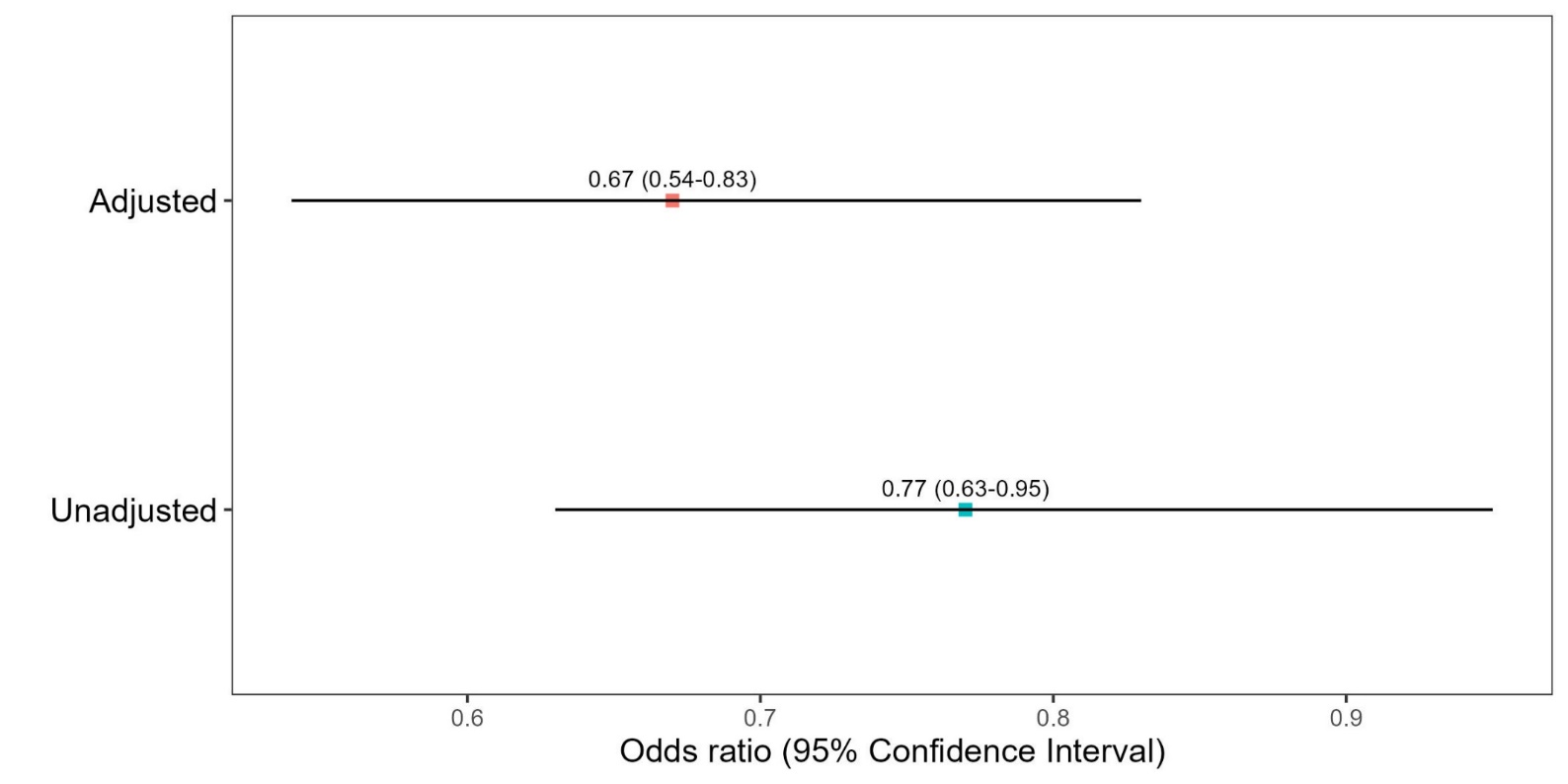


^a^ Updated matched dataset includes matching on hospital and hospital unit in addition to matching criteria mentioned in Methods section.

#
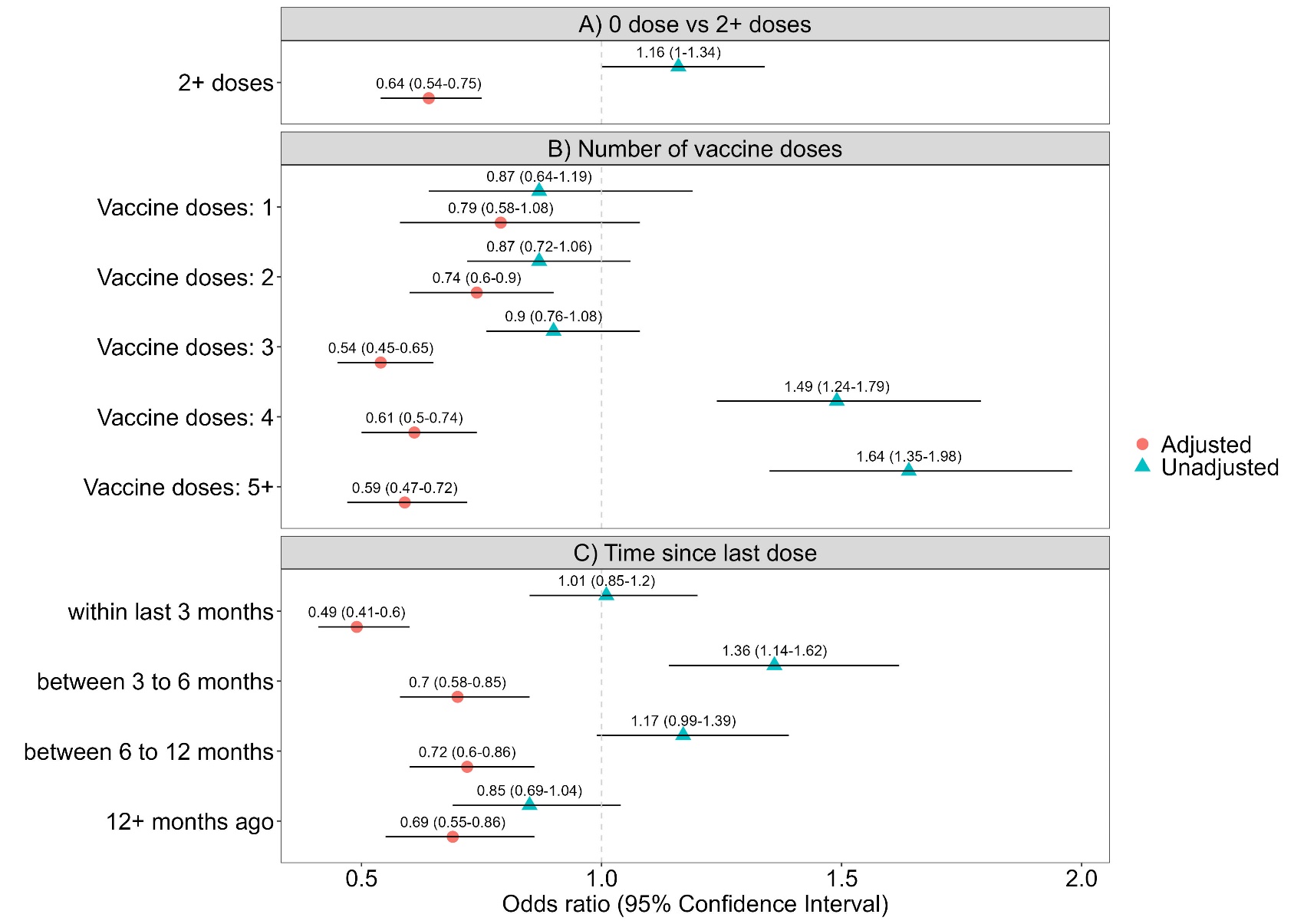
eFigure2: Odds ratio for association between vaccination and nosocomial SARS-CoV-2 infection comparing cases and controls including patients with 1 vaccine dose

A) comparing patients with 0 or 1 vaccine doses to those with 2+ vaccine doses

B) comparing patients with 0 vaccine doses to those with 1, 2, 3, 4, 5+ vaccine doses

C) comparing patients with 0 or 1 vaccine doses to those with 2+ vaccine doses such that the last vaccine dose was administered within last 3 months, between 3 to 6 months, between 6 to 12 months and 12+ months ago.

All patients had their vaccine dose counted until 14 days prior to index date to account for development of

complete immune response post vaccination. Analyses were restricted to period 1 and 2.
